# Supplementary material for: Lipidomic Profiling Identifies Signatures of Poor Cardiovascular Health
Source: Metabolites. 2021 Oct 29;11(11):747. doi: 10.3390/metabo11110747 (PMC8624456; doi:10.3390/metabo11110747)

Table S1. Distribution of CVH metrics among 461 Kardiovize participants

CVH metric	Poor	Intermediate	Ideal
**Smoking status**	18.0%	27.3%	54.7%
**Healthy dietary score**	3.3%	96.7%	-
**Physical activity**	15.1%	38.0%	46.9%
**BMI**	10.0%	41.2%	40.8%
**Blood pressure**	19.8%	43.8%	36.4%
**Total cholesterol**	14.9%	42.1%	43.0%
**Fasting glucose**	4.7%	16.6%	78.7%

The parameters for each CVH metric are described in Table S7.

Table S2. Important lipid features identified by ANOVA analysis and Fisher's least significant difference method across CVH categories of smoking status

Lipid species	F-statistic	*P*-value	FDR
**LPE 22:6**	6.4857	0.001669	0.049792
**PC 14:1_14:1**	6.4227	0.001774	0.049792
**LPE 16:0**	6.293	0.002013	0.049792
**PE 18:2_16:1**	6.2086	0.002186	0.049792
**PE 18:0_18:0**	6.0808	0.002475	0.049792
**LPE 20:5**	5.9263	0.002877	0.049792

Table S3. Important lipid features identified by ANOVA analysis and Fisher's least significant difference method across CVH categories of blood pressure

Lipid species	F-statistic	*P*-value	FDR
**PE 18:0_20:5**	11.694	1.11E-05	0.001038
**PE 18:0_22:4**	10.727	2.80E-05	0.001038
**SM d18:1/18:1**	10.655	3.00E-05	0.001038
**PE 16:0_18:1**	10.396	3.84E-05	0.001038
**PE 16:0_16:1**	10.116	5.02E-05	0.001085
**PC 18:0_20:5**	9.4165	9.82E-05	0.001768
**PE 18:0_16:0**	8.9346	0.000156	0.002408
**PE 18:1_20:1**	8.7966	0.000178	0.002408
**PE 16:0_20:2**	8.3629	0.000271	0.002926
**PE 16:0_20:3**	8.3627	0.000271	0.002926
**PE 18:1_22:6**	8.039	0.00037	0.003636
**LPE 20:5**	7.9369	0.000409	0.003679
**LPC 20:5**	7.7266	0.000501	0.004161
**PE 16:0_18:2**	7.5371	0.000602	0.004642
**LPE 20:4**	7.1707	0.000858	0.006179
**SM d18:1/14:0**	6.9202	0.001094	0.007305
**PC 18:0_20:3**	6.8691	0.00115	0.007305
**PE 16:0_20:1**	6.7923	0.001239	0.007434
**LPE 20:3**	6.7106	0.001341	0.007624
**SM d18:1/20:1**	6.5796	0.001523	0.008199
**PC 16:0_20:5**	6.5328	0.001594	0.008199
**PE 18:0_18:0**	6.2402	0.002119	0.010403

**PC 18:0_20:0**	5.9361	0.00285	0.013196
**PE 18:1_22:4**	5.9068	0.002933	0.013196
**PE 18:2_20:5**	5.7038	0.003575	0.01506
**PC 18:0_20:2**	5.6279	0.003849	0.01506
**PE 18:2_16:1**	5.6185	0.003885	0.01506
**PC 18:1_20:5**	5.608	0.003925	0.01506
**PC 16:0_18:0**	5.5774	0.004044	0.01506
**SM d18:1/16:0**	5.5117	0.004312	0.015523
**SM d18:1/18:0**	5.4601	0.004535	0.015798
**PC 16:0_16:1**	5.2998	0.005304	0.0179
**PC 18:0_18:0**	5.1826	0.005947	0.01894
**Cer d18:1/14:0**	5.1543	0.006114	0.01894
**SM d18:1/20:0**	5.1238	0.006299	0.01894
**PC 18:0_22:4**	5.1215	0.006314	0.01894
**PE 18:2_20:1**	5.0932	0.006491	0.018947
**PC 16:0_20:2**	4.8536	0.008206	0.023323
**SM d18:1/22:1**	4.8123	0.008545	0.023662
**LPE 20:2**	4.7077	0.009467	0.025561
**LPE 18:3**	4.5268	0.011304	0.029776
**LPE 16:1**	4.4466	0.012229	0.030746
**PC 16:0_18:1**	4.435	0.012368	0.030746
**LPC 22:6**	4.4221	0.012526	0.030746
**PE 18:2_20:4**	4.277	0.014443	0.034664
**SM d18:1/24:1**	4.2298	0.015127	0.035517
**PE 16:0_16:0**	4.0991	0.0172	0.039523
**LPE 22:5**	4.0018	0.018926	0.042584
**SM d18:1/26:1**	3.9074	0.020767	0.045652
**PC 18:1_22:5**	3.8704	0.021535	0.045652
**PC 14:1_14:1**	3.8693	0.021558	0.045652
**LPE 18:0**	3.8001	0.023076	0.047927
**SM d18:1/24:0**	3.7769	0.02361	0.048111

Table S4. Important lipid features identified by ANOVA analysis and Fisher's least significant difference method across CVH categories of total cholesterol

**Lipid species**	**F-statistic**	***P*-value**	**FDR**
**PC 18:0_20:5**	11.295	1.63E-05	0.000905
**PE 18:0_20:5**	11.265	1.68E-05	0.000905
**Cer d18:1/14:0**	9.987	5.69E-05	0.002047
**PC 16:0_20:5**	7.1388	0.000885	0.023903
**PE 18:1_22:6**	6.6916	0.001367	0.029516

Table S5. Important lipid features identified by ANOVA analysis and Fisher's least significant difference method across CVH categories of blood fasting glucose

Lipid species	F-statistic	*P*-value	FDR
**PE 18:1_22:6**	7.5593	0.000589	0.026949
**PE 18:0_20:5**	7.1709	0.000858	0.026949
**PE 18:0_22:4**	6.9553	0.001058	0.026949
**LPE 20:3**	6.7171	0.001333	0.026949
**LPE 20:4**	6.5234	0.001609	0.026949
**PC 16:0_20:5**	6.4964	0.001652	0.026949
**PC 18:0_20:5**	6.4391	0.001747	0.026949
**PC 18:1_20:5**	6.2336	0.002133	0.028798
**LPE 20:2**	5.9228	0.002888	0.033241
**PE 18:1_20:1**	5.8573	0.003078	0.033241
**PE 16:0_18:1**	5.5919	0.003988	0.033887
**PE 18:1_22:4**	5.4416	0.004618	0.033887
**PE 18:2_20:1**	5.4217	0.004709	0.033887
**PE 16:0_16:1**	5.3999	0.00481	0.033887
**PE 16:0_20:3**	5.321	0.005195	0.033887
**PE 18:0_18:0**	5.2357	0.005647	0.033887
**Cer d18:1/14:0**	5.2053	0.005817	0.033887
**PC 18:0_18:0**	5.2002	0.005846	0.033887
**PE 16:0_20:2**	5.1803	0.005962	0.033887

Table S6. Important lipid features identified by ANOVA analysis and Fisher's least significant difference method across categories of CVH

Lipid species	F-statistic	*P*-value	FDR
**PE 18:0_20:5**	14.581	7.27E-07	5.05E-05
**PE 18:0_22:4**	14.154	1.09E-06	5.05E-05
**PE 16:0_16:1**	13.882	1.40E-06	5.05E-05
**PC 18:0_20:5**	11.423	1.44E-05	0.000389
**PE 16:0_20:3**	10.468	3.59E-05	0.000703
**PE 16:0_18:1**	10.379	3.91E-05	0.000703
**PC 16:0_20:5**	9.7656	7.03E-05	0.001085
**PC 16:0_16:1**	9.4294	9.71E-05	0.001311
**LPE 20:3**	8.6153	0.000212	0.002383
**SM d18:1/18:1**	8.5758	0.000221	0.002383
**PE 18:1_22:6**	8.3384	0.000277	0.002686
**PE 18:1_22:4**	8.1654	0.000328	0.002686
**PC 18:0_20:3**	8.1489	0.000333	0.002686
**PE 16:0_18:2**	8.103	0.000348	0.002686
**PC 18:1_20:5**	7.4499	0.000655	0.004715
**LPC 20:5**	7.3316	0.000734	0.004778
**PE 18:0_16:0**	7.3071	0.000752	0.004778
**PC 18:0_22:4**	7.209	0.000827	0.004962
**PE 18:0_18:0**	7.1407	0.000884	0.005023
**SM d18:1/14:0**	7.0697	0.000947	0.005112

**PE 16:0_20:2**	7.0128	0.001001	0.005145
**LPE 20:4**	6.5918	0.001506	0.007391
**PE 18:1_20:1**	6.3393	0.001925	0.009038
**LPE 18:3**	6.2436	0.002113	0.009507
**PC 18:0_20:2**	6.0582	0.002531	0.010932
**PE 16:0_16:0**	5.918	0.002901	0.011818
**LPE 16:1**	5.8982	0.002958	0.011818
**PE 18:2_20:5**	5.862	0.003064	0.011818
**LPE 18:1**	5.6719	0.003688	0.013735
**PC 18:0_20:0**	5.5107	0.004317	0.015228
**PE 18:2_16:1**	5.4706	0.004489	0.015228
**LPE 20:2**	5.4654	0.004512	0.015228
**PC 16:0_22:4**	5.4051	0.004785	0.015661
**PC 18:1_22:4**	5.1196	0.006326	0.019653
**PC 16:0_20:2**	5.1127	0.006369	0.019653
**PE 18:2_20:4**	5.0349	0.006872	0.020312
**PE 18:2_20:1**	5.0221	0.006959	0.020312
**PC 16:0_18:0**	4.9842	0.007222	0.020526
**PC 18:1_22:5**	4.7424	0.009152	0.025343
**PC 16:1_18:2**	4.6256	0.010261	0.027706
**PC 18:0_18:0**	4.5757	0.010775	0.028374
**PC 16:0_18:1**	4.5515	0.011034	0.028374
**PC 18:1_18:3**	4.4249	0.012492	0.031376
**PE 18:2_22:6**	4.3549	0.01338	0.032843
**SM d18:1/24:1**	4.3245	0.013787	0.033088
**LPC 22:4**	4.2517	0.014807	0.034764
**PE 16:0_20:1**	4.2147	0.015355	0.035182
**PC 18:2_20:5**	4.1962	0.015636	0.035182
**LPC 20:4**	4.171	0.016029	0.035329
**LPE 18:2**	4.1044	0.017112	0.036962
**SM d18:1/16:0**	3.9572	0.019775	0.041876
**LPC 22:5**	3.8649	0.021654	0.044174
**SM d18:1/18:0**	3.8553	0.021858	0.044174
**LPC 20:3**	3.8448	0.022087	0.044174
**LPC 16:1**	3.785	0.023424	0.045997
**PE 18:2_18:2**	3.6614	0.026454	0.049626
**LPC 20:2**	3.6559	0.026596	0.049626
**LPE 20:5**	3.6538	0.026651	0.049626
**LPC 22:6**	3.6347	0.027159	0.049714

Table S7. Definition of cardiovascular health metrics.

Health Metric		Definition
Smoking	Ideal<br>Intermediate<br>Poor	Never or quit > 12 months<br>Former ≤ 12 months<br>Current
Body mass index	Ideal<br>Intermediate<br>Poor	< 25kg/m^2<br>25-29.9 kg/m^2<br>≥ 30 kg/m^2
Physical Activity	Ideal<br><br>Intermediate<br><br>Poor	≥ 150 min/week moderate, ≥ 75 min/week vigorous or ≥ 150 min/week moderate + vigorous<br>1–149 min/ week moderate or 1–74 min/week vigorous or 1–149 min/week moderate + vigorous<br>None
Fasting serum glucose	Ideal<br>Intermediate<br>Poor	< 5.55 mmol/l, without treatment<br>5.55-6.94 mmol/l or treated to 5.5 mmol/l<br>≥ 6.95 mmol/l
Total cholesterol	Ideal<br>Intermediate<br>Poor	< 5.17 m mol/l without medication<br>5.17-6.18 mmol/l, or treated to < 5.17 mmol/l<br>≥ 6.19 mmol/l
Blood Pressure	Ideal<br>Intermediate<br>Poor	SBP < 120 and DBP < 80 mmHg, without medication<br>SBP 120-139 or DPB 80-89 mmHg, or treated to hypertension<br>SBP ≥ 140 or DBP ≥ 90 mmHg
Healthy diet score	Ideal<br>Intermediate<br>Poor	4-5 components<br>2-3 components<br>0-1 component
	**COMPONENTS:**	A) ≥ 4,5 cups/day of fruits and vegetables
		B) ≥ 2 servings/week of fish
		C) ≥ 3 servings/day of whole grains
		D) < 1500 mg/day of sodium
		E) 450 kcal (or 1 litre)/week of sweet/sugar sweetened beverages

Medina-Inojosa JR, Vinciguerra M, Maugeri A, Kunzova S, Sochor O, Movsisyan N, Geda YE, Stokin GB, Lopez-Jimenez F. Prevalence of ideal cardiovascular health in a Central European community: results from the Kardiovize Brno 2030 Project. Eur J Prev Cardiol. 2020; 27(4):441-443.

Figure S1. ANOVA analysis of lipid species across CVH categories of smoking status. Lipid species with adjusted *P*-value (FDR) <0.05 are indicated in red.

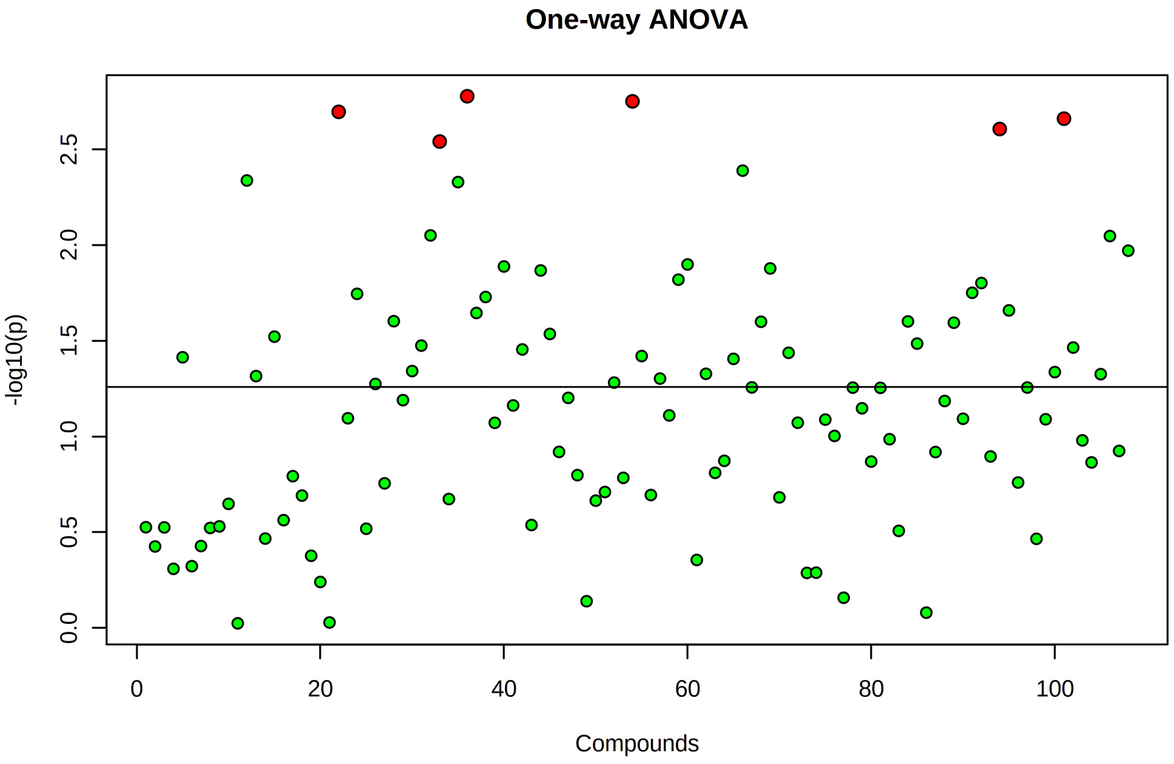

Figure S2. ANOVA analysis of lipid species across CVH categories of physical activity.

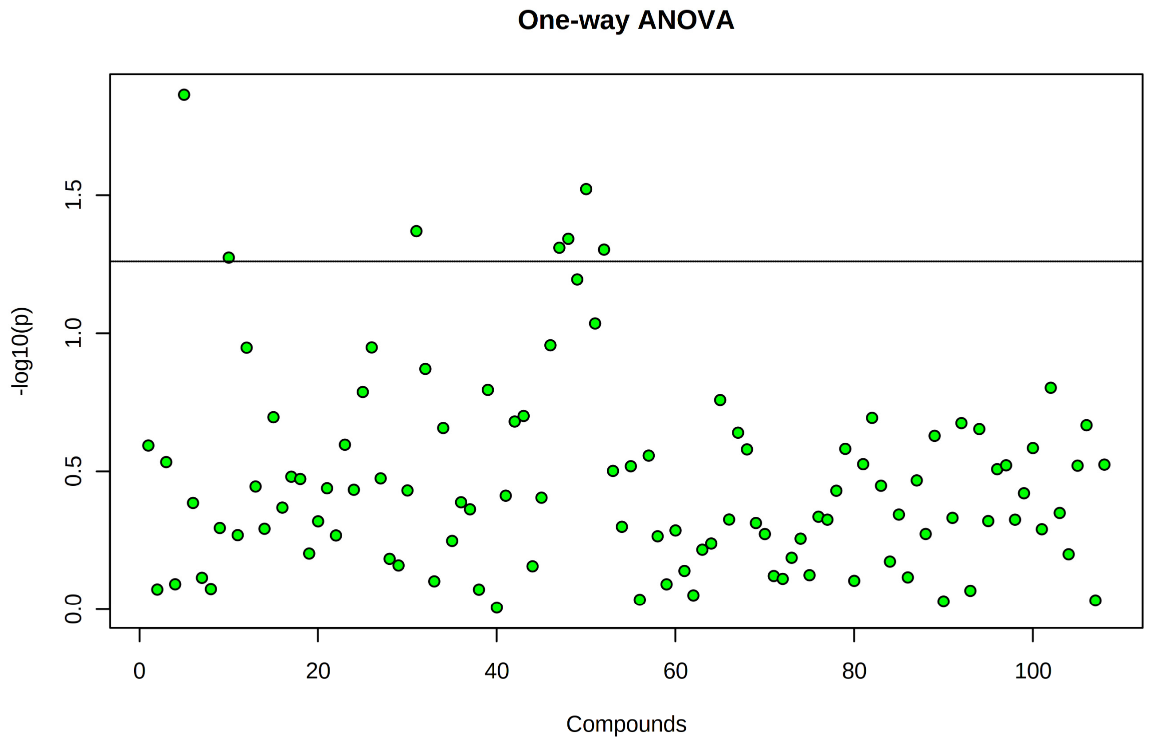

Figure S3. ANOVA analysis of lipid species across CVH categories of BMI.

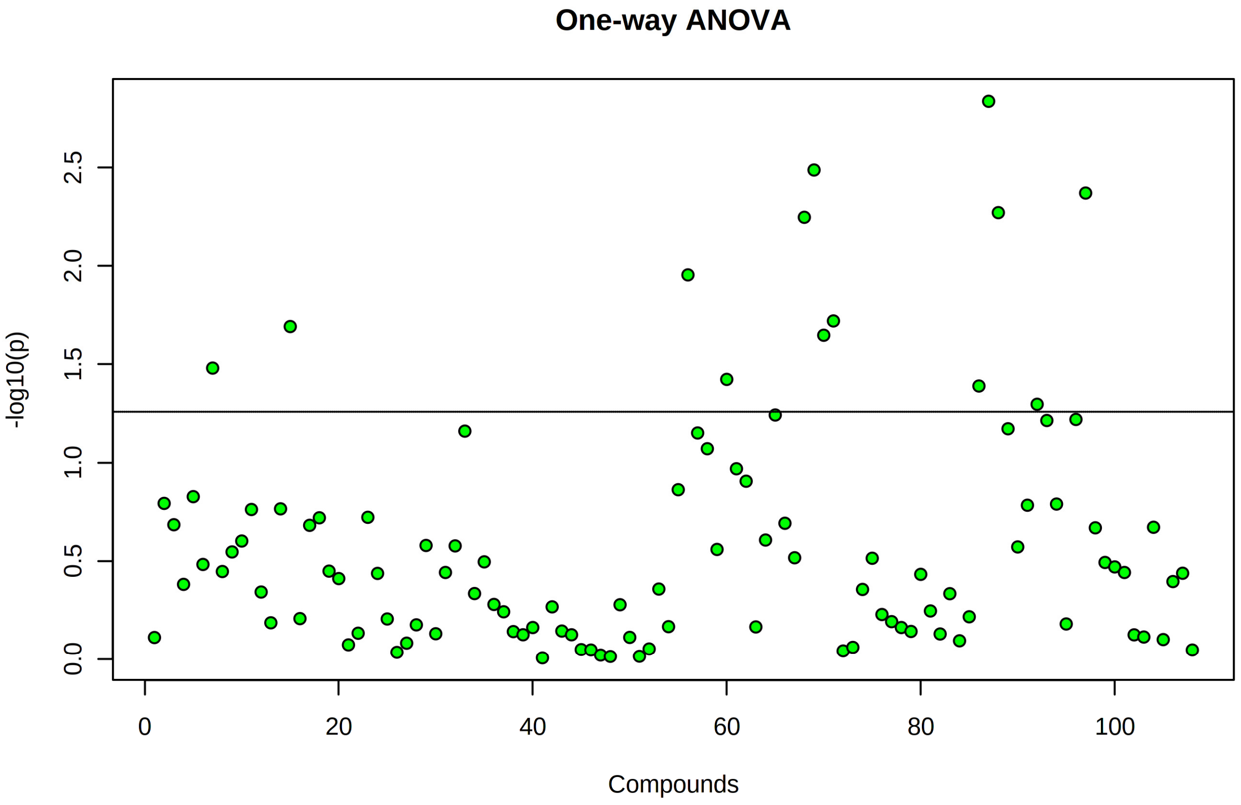

Figure S4. ANOVA analysis of lipid species across CVH categories of blood pressure. Lipid species with adjusted *P*-value (FDR) <0.05 are indicated in red.

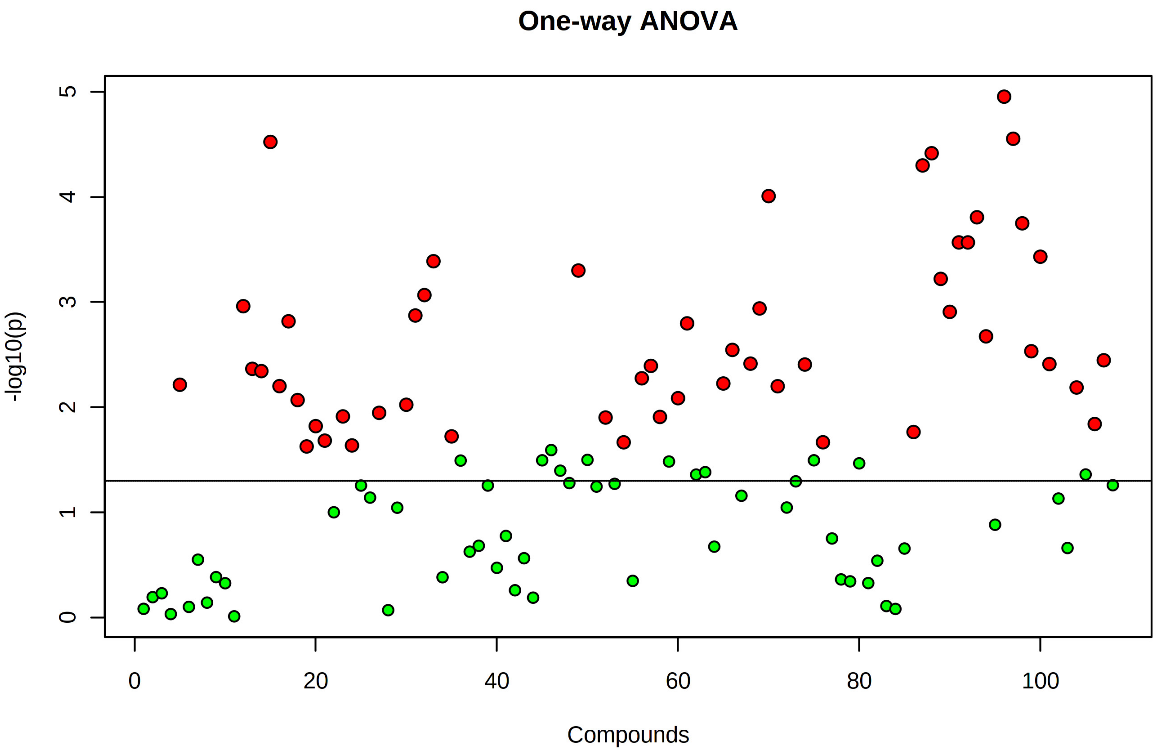

Figure S5. ANOVA analysis of lipid species across CVH categories of total cholesterol. Lipid species with adjusted *P*-value (FDR) <0.05 are indicated in red.

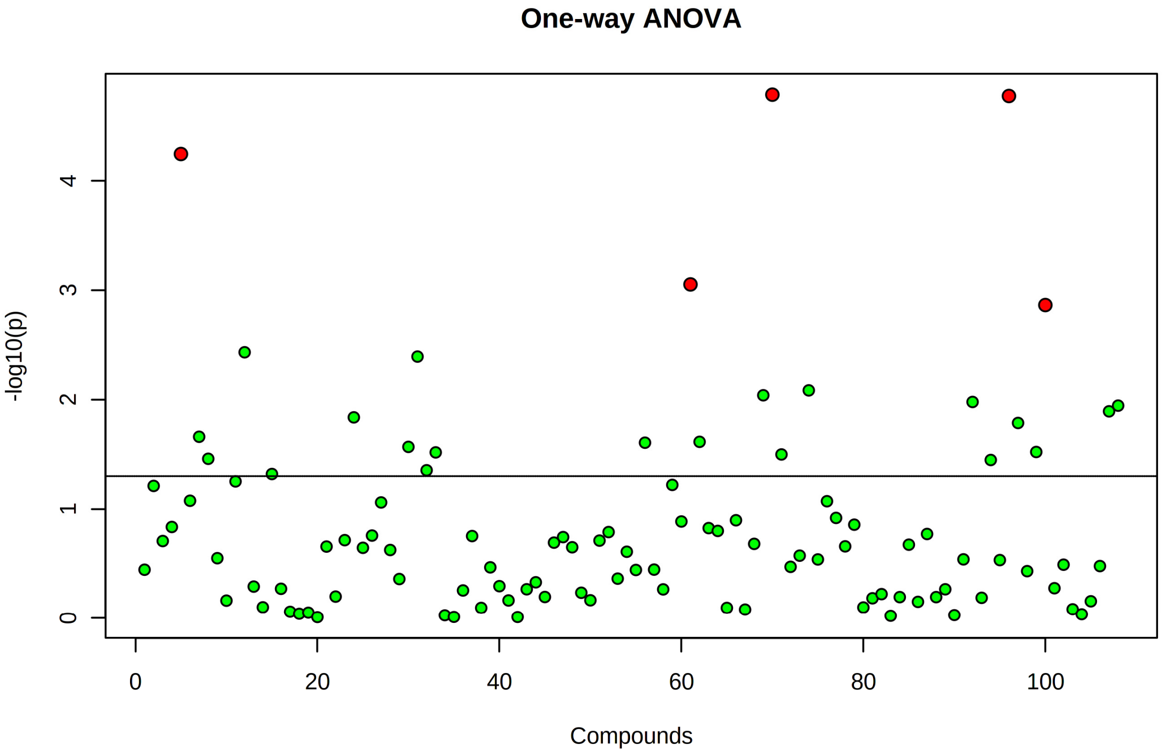

Figure S6. ANOVA analysis of lipid species across CVH categories of blood fasting glucose. Lipid species with adjusted *P*-value (FDR) <0.05 are indicated in red.

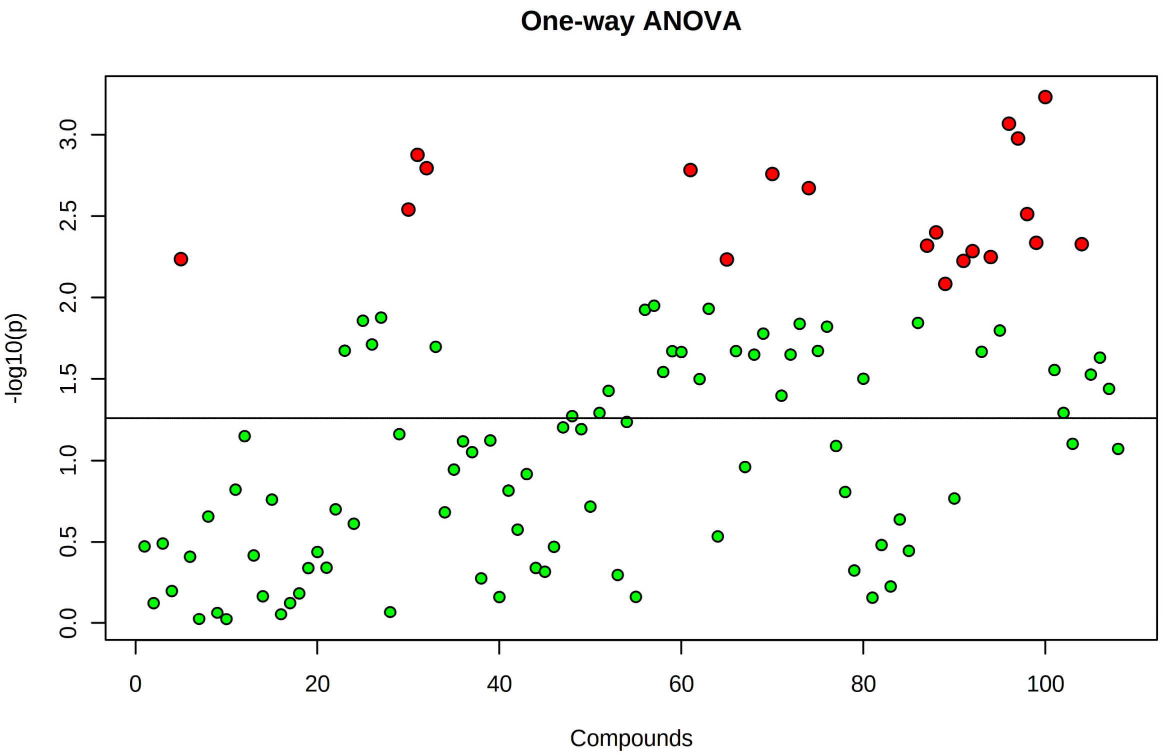

Figure S7. ANOVA analysis of lipid species across categories of CVH. Lipid species with adjusted *P*-value (FDR) <0.05 are indicated in red.

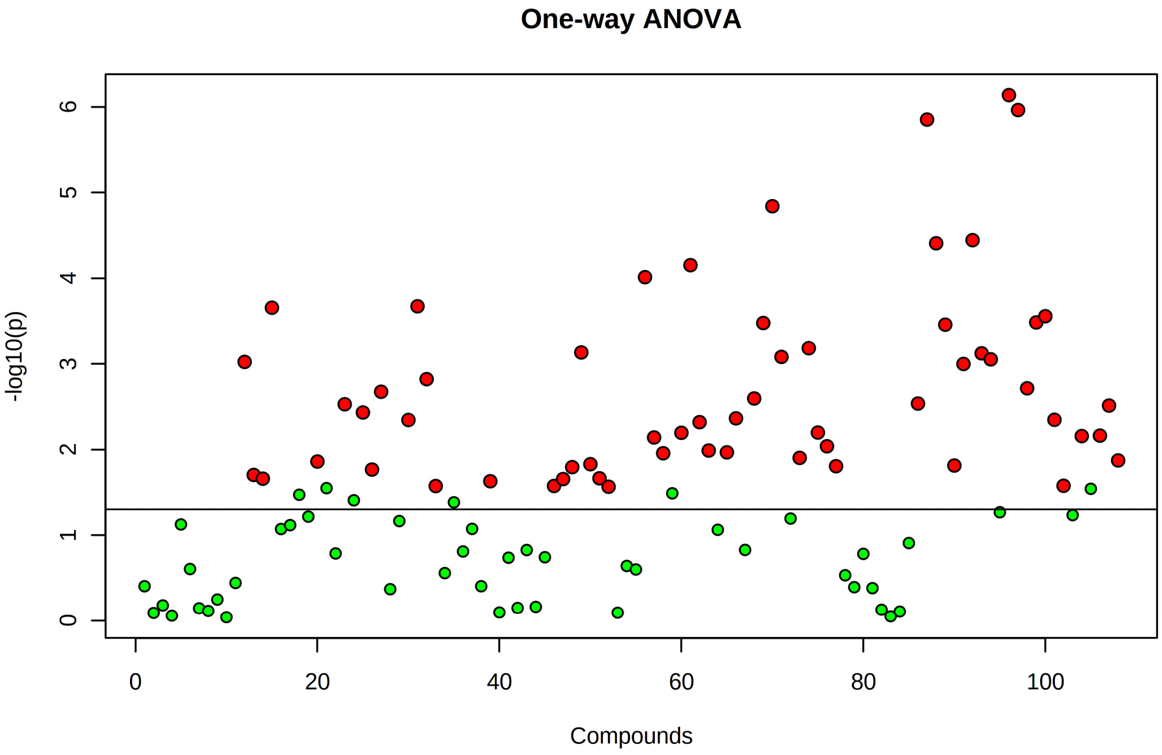

Figure S8. Classification performance and permutation test of the PLS-DA. (A) PLS-DA classification using different number of components. The red star indicates the best classifier. (B) PLS-DA model validation by permutation tests based on separation distance.

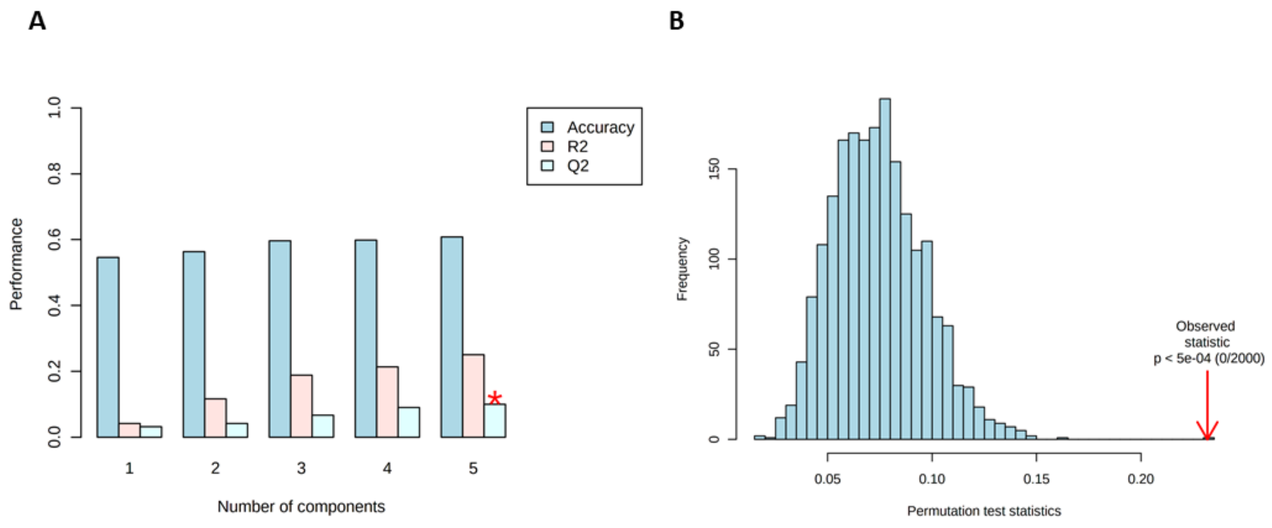

Supplement: Supplementary file 1 [file metabolites-11-00747-s001.zip › metabolites-1436312-supplementary.pdf]
